# Intestinal Obstruction, with Special Reference to the Duty of the General Practitioner*Read before the East Texas Medico-Chirurgical Association at Crockett, Texas, December 2, 1904.

**Published:** 1905-07

**Authors:** W. C. Lipscomb

**Affiliations:** Crockett, Texas


For Texas Medical Journal.

### Intestinal Obstruction, With Special Reference to the Duty of the General Practitioner.*

BY W. C. LIPSCOMB, M. D., CROCKETT, TEXAS.

Careful attention to the caption of this paper will indicate that no attempt is made to go exhaustively into the etiology and surgical treatment of this class of cases, but rather to call the attention to salient points to be considered in order that conservative measures may be promptly adopted. I make no claim to having made an exhaustive study of the subject. My experience has been too limited and not one to prompt me to approach the subject with any degree of confidence. It is one of the most difficult and distressing and at the same time one of the most important problems that confront us; especially apart from the centers of population, where expert specialism is not promptly available.

It is then, for these reasons, that I desire to present this subject to your consideration, hoping that free discussion may evolve something of practical value to us all. To be practical and briefly to the point, I will avoid an effort at systematic classification.

When called to a case of obstruction, the first question we should

---

*Read before the East Texas Medico-Chirurgical Association at Crockett, Texas, December 2, 1904.

seek to solve is as to whether it is a case fairly promising under palliative measures, or is it a case for prompt surgical interference? This question taxes our powers of discrimination and, perhaps, our moral courage to the utmost. Symptoms are common to many conditions, surgical and non-surgical, and conclusions are difficult. If we at last settle the question satisfactorily to our own minds, we may meet with another difficulty in the patient who persistently refuses to regard his case as a surgical one. Then if there is an early operation and it proves unsuccessful, the operation is held accountable for what was inevitable. The conscientious physician, however, is never deterred by ignorant criticisms.

In reaching our conclusions we should remember that bowel stasis is the result always of one of three conditions, viz.: paralytic obstruction, mechanical obstruction, and peritonitis.

The first of these as a disease per se, like many other nervous diseases, is obscure in its origin and hopeless of relief. Fortunately it is rare. As a secondary condition it may appear as a sequel either of peritonitis or of mechanical obstruction; so that practically we have only the last two diseases to consider.

It must be borne in mind that surgical treatment is the ultimatum in both affections. Therefore, the diagnosis should be promptly made and opiates withheld so as not to obscure it.

Preceding the development of peritonitis we have a history of appendicular trouble—typhoid fever, intestinal catarrh, rheumatism, gonorrhea, and traumatism, penetrating or contusing; also frequently chill and high fever. If now effusion can be detected in advance of meteorism, the diagnosis is established as differentiated from mechanical obstruction. Muscular rigidity over the entire abdomen, general abdominal tenderness and a desire to lie quiet, as contrasted with a disposition in the other disease to move about mark peritonitis. Pain and temperature are negative usually. Pulse may have the classic whip-cord character, but this is not constant. "Breathing is characteristic, thoracic shallow and frequent" (Douglas). Position first on back, later partly on side, always so as to relieve tension of abdominal muscles. Intensely septic cases present pale and mottled skin over abdomen. Vesicles and pustules here indicate a fatal issue.

In mechanical obstruction, by far the most frequent form is fecal impaction. A history of constipation, muscular atony, etc., absence of acute symptoms at the outset, tumor with doughy feel, sallow complexion, indicate this. Treatment is by high enemas; the sooner the better the prognosis. It must be borne in mind that this sometimes fails, and the indication is then surgical. After immediate relief, long continued treatment for muscular atony is indicated.

Parasites rarely occlude the intestine. When discoverable, gentle massage of bowels, anthelmintics and high enemas will usually suffice. When there is history of foreign body swallowed, the nature of the substance and condition of patient are to be considered, and, if promising, palliative measures employed.

A history of hepatic colic and other bilious signs may suggest impaction of gall stones. These will usually yield to high enemas. If not, enterotomy is employed.

The above, according to Douglas, constitute principally the chronic forms of mechanical obstruction in which palliative measures are usually effective. It is to be remembered that all forms of occlusion are acute in their final manifestations.

Douglas's classification of the acute causes are:

1. Through congenital apertures.
2. Into normal peritoneal fossæ.
3. Through mesenteric slits and diaphragmatic rents.
4. By bands congenital or adventitious.
5. By peritoneal adhesions.
6. Vitelline remains.
7. About viscera abnormally attached.
8. Kinks and knots.
9. Volvulus.
10. Intussusception.

A differential consideration of all these causes and the intricate and ingenious symptomology they present, would carry this paper entirely beyond its scope.

In conclusion, I will say that in both peritonitic and mechanical obstruction, surgical interferences is indicated where palliative measures fail. First, if no other complication preclude recovery; second, if shock intervenes and can be counteracted partially and a reactionary condition maintained. Symptoms of shock come on so gradually and treacherously in some cases that unless our patient is closely watched and the first symptoms noted, we may lose valuable time. In cases suspected to be of this class, the rate and character of the pulse should be noted closely and often, and the abdomen carefully gone over for the first physical sign. We can not be too watchful. We should be well provided with means to carry out such measures as will accomplish the conservation of vitality and reaction from shock. To quote from Douglas, "Intravenous injection of normal saline solution, alcohol, strychnia, and rectal alimentation are the remedies demanded."

